# Typhoid Fever Diagnosis in Endemic Countries: A Clog in the Wheel of Progress?

**DOI:** 10.3390/medicina54020023

**Published:** 2018-04-25

**Authors:** Olumide Ajibola, Mari B. Mshelia, Bashar H. Gulumbe, Anthonius A. Eze

**Affiliations:** 1 Department of Microbiology, Faculty of Science, Federal University Birnin Kebbi, P.M.B. 1157 Kalgo, Kebbi State, Nigeria; bata.mari@fubk.edu.ng (M.B.M.); hgbashar@gmail.com (H.B.G.); 2 Department of Medical Biochemistry, University of Nigeria, Enugu Campus, Enugu 400241, Nigeria

**Keywords:** Typhoid fever, S. Typhi, serodiagnosis, multidrug resistance, laboratory

## Abstract

Typhoid fever causes significant morbidity and mortality in developing countries, with inaccurate estimates in some countries affected, especially those situated in Sub-Saharan Africa. Disease burden assessment is limited by lack of a high degree of sensitivity and specificity by many current rapid diagnostic tests. Some of the new technologies, such as PCR and proteomics, may also be useful but are difficult for low-resource settings to apply as point-of-care diagnostics. Weak laboratory surveillance systems may also contribute to the spread of multidrug resistant *Salmonella* serovar Typhi across endemic areas. In addition, most typhoid-endemic countries employ serological tests that have low sensitivity and specificity making diagnosis unreliable. Here we review currently available typhoid fever diagnostics, and advances in serodiagnosis of S. Typhi.

## 1. Introduction

“Typhoid fever” (TF) was coined by the French physician, Pierre Charles Alexander Louis, who gave a description of the clinical signs and symptoms of the disease to be typhoidal, with signs of mental fogginess and persistent fever which mimicked the symptoms caused by typhus [1,2]. TF is a systemic infection comprising of diseases caused by *Salmonella enterica* serovar Typhi; while *S. enterica* serovar Paratyphi (A, B and C) cause paratyphoid fever, other serovars of *Salmonella* are grouped as non-typhoidal. S. Typhi belongs to the family Enterobacteriaceae, which are Gram-negative rods and facultative anaerobes. The genus *Salmonella* is classified into two species, *S. enterica* and *S. bongori*, and more than 2600 serovars (or serotypes), based on its lipopolysaccharide (LPS) cell wall (somatic O antigen), the flagella (H antigen) and its surface Vi antigen (present only in S. Typhi, *S. paratyphi C*, *Citrobacter freundii*, and *S. dublin*) [3]. S. Typhi are restricted exclusively to human hosts and are associated with systemic infection, prolonged fever and may result in an asymptomatic carrier state. Following resolution of infection, a small number of persons, called carriers, continue to carry the bacteria. Around 2–5% of those who contract typhoid fever become chronic carriers, as the bacteria persists in the biliary tract after symptoms have resolved [4]. Seroepidemiological surveys have also revealed that some persons in the population have non-immunising antibodies present in them irrespective of previous vaccination status, and will be seropositive, which is a limitation of employing serodiagnostic rapid diagnostic tests (RDTs) in surveillance [5]. TF was an important cause of illness and death in the United States and Europe in the 19th century, as a result of overcrowded and unsanitary conditions. The provision of treated municipal water, pasteurization of dairy products and better sanitary conditions led to a steep decline in the incidence of typhoid in these regions [3,6]. In most developing countries, however, access to safe drinking water and adequate sanitation remains a challenge. TF causes significant morbidity and mortality especially in Asia, Africa (although evidence suggests that the impact might be under-appreciated), Latin America and the Middle East. Despite the availability of vaccines, vaccination against TF remains limited in most of the affected countries. Lack of inclusion of typhoid vaccines into routine immunization programs in addition to poor TF surveillance and weak laboratory infrastructure in most countries affected further aggravates morbidity. TF is essentially a disease of the poor, uneducated and usually the most vulnerable in the society who lack access to basic social services such as health care, good hygiene and safe drinking water. Emergence of antibiotic resistance (ABR) strains of S. Typhi to fluoroquinolones has made management of TF in endemic countries even more challenging.

In most TF affected countries, physicians rely on laboratory results from serologic tests that are not reliable (usually Widal) to arrive at a clinical decision. Application of sensitive and specific diagnostic tests in health facilities, and establishment of active surveillance programs in endemic regions is important for accurate antibiotic prescriptions against local S. Typhi strains in circulation and detection of emerging ABR strains in the population. Here, we review laboratory diagnosis of TF, emergence of ABR S. Typhi and propose solutions to challenges encountered in TF-endemic countries.

## 2. Disease Burden

TF was estimated to cause approximately 21.65 million illnesses and 216,000 deaths worldwide in the year 2000 [7]. In 2010, the estimated number of cases after adjusting for water-related risk factors was 11.9 million cases and 129,000 deaths [7]. Studies report that TF is more prevalent in Asia; high-risk groups are children and infants, while those at risk of complicated TF include neonates and pregnant women [8,9]. In some countries in Asia, *Salmonella* *enterica* serovar Paratyphi A has accounted for a growing proportion of enteric fever; however, this is not the focus of this review [10].

## 3. Source and Mode of Transmission

Transmission of Typhoidal *Salmonella* is primarily through the fecal–oral route, i.e., through water or food contaminated with human feces. Contaminated water sources have also resulted in large epidemics of the disease [11,12]. TF is mainly found in developing countries, and it has been eradicated from developed countries through good hygiene, sanitary and availability of potable drinking water [13,14]. Poor hygiene practices by food handlers who usually lack good hygienic practices also encourages TF transmission in developing countries.

## 4. Pathogenesis

The minimum infectious dose of S. Typhi could be as low as 1000 or as high as a million bacteria based on studies carried out in human volunteers [15]. Following ingestion of the bacteria, it binds to mucosa cells in the small intestine, eventually invading the mucosa. Post invasion of the mucosa, the bacteria are translocated to the lymphoid follicles draining mesenteric lymph nodes, and some find their way into the reticuloendothelial cells of the liver and spleen. The bacteria are phagocytosed and have the ability to survive and multiply within macrophages of the lymphoid follicles, liver and spleen. Once the bacteria multiply to a certain threshold, in addition to their virulence and the host response, bacteria are released and sequestered into the blood stream. Following release into the bloodstream, they invade secondary sites such as the Peyer’s patches of the terminal ileum, liver, spleen, bone marrow or the gall bladder [3]. Bacteria that are excreted in the bile re-invade the intestinal wall or are passed on through the feces. Cell counts of patients with acute TF gave a median bacteria concentration of 1 bacterium per ml of blood (about 66 percent of which are inside phagocytic cells) and about 10 bacteria per ml of bone marrow [16]. This indicates that the bone marrow has a higher concentration of bacteria and is a better site than blood for detection of S. Typhi in patients. S. Typhi infection has been shown to induce local and systemic immune responses in humans, but this provides incomplete protection against relapse and reinfection [17].

## 5. Laboratory Diagnosis of Typhoid Fever

Isolation of the causative bacteria in TF patients by culture remains the gold standard for diagnosis. Culture is the most reliable way of detecting typhoid in infected patients, and usually by blood culture, but bone marrow culture has a greater sensitivity. However, in most developing countries a serological test known as the Widal test is most commonly applied. Below we discuss the most commonly used laboratory approaches in typhoid detection in endemic countries and the challenges attributed to each (Table 1).

## 6. Bacteria Culture

The definitive diagnosis of TF is isolation of S. Typhi from blood which should normally be sterile. Blood samples can be cultured on an enriched media (Blood agar) and a differential media (MacConkey agar). S. Typhi is a non-lactose fermenter producing smooth pale colonies on selective media. S. Typhi utilize citrate as carbon source, lysine as nitrogen source and produce hydrogen sulphide which is responsible for the characteristic black centers on *Salmonella-Shigella* agar or black butt with no gas on Kliger iron agar [40]. The volume of blood collected from patients is one of the key factors in isolation of S. Typhi. Ideally 10–15 mL of blood should be collected from school children and adults, while toddlers and preschool children supply 2–4 mL of blood. Toddlers and preschool children require less blood because they have higher bacteremia than adults. The optimum ratio of blood to culture media for isolation of pathogens is 1:10. Blood specimens are inoculated into Brain heart infusion broth or tryptone soy broth immediately, incubated at 37 °C and subcultured at days 1, 2, 3 and 7 on blood agar (horse or sheep blood) or MacConkey agar and incubated at 37 °C for 18–24 h. Blood agar allows the growth of both Gram-positive and -negative pathogens, while MacConkey selects for Gram-negative pathogens only. In patients who have yet to initiate antibiotic treatment, within the first two weeks of infection, blood culture is positive in up to 80% of patients, while in patients already on antibiotics, sensitivity can be as low as 40% [41]. In patients already on antibiotics the preferred sample to be collected is the bone marrow, because there is a preponderance of bacteria at this site, where they multiply and reside. Blood samples for culture should be collected within 7–14 days, as bacteria counts in blood decline as disease progresses. The volume of blood collected is also a critical factor to consider in order to obtain positive results, because 1 mL of blood contains <10 bacteria [16,42,43,44]. In contrast, bone marrow culture is more sensitive giving good yields even when antibiotic treatment has been initiated. The reliability of bone marrow samples for culture is directly linked to a higher bacteria count in the sample. Isolation of the causative bacteria in culture makes it possible to carry out further testing such as antimicrobial susceptibility testing to determine the best antibiotic to be prescribed by the clinician, identification of multidrug resistant strains, epidemiologic typing and molecular characterization.

In resource-constrained settings, isolation of S. Typhi from stool/rectal swab culture which is more routinely used in most diagnostic laboratories is suggestive of typhoid fever. Stool samples should be collected in sterile wide-mouthed containers, and the quantity supplied by the patient directly affects the likelihood of isolation of S. Typhi. In the absence of stool samples, rectal swabs inoculated in Carry Blair transport medium (Thermo Scientific, Loughborough, UK) may suffice, though with limited success. Stool samples should be inoculated within 2 h of collection or stored at 4 °C until ready for inoculation. Approximately 1 g of stool sample is inoculated into 10 mL of Selenite F broth, 37 °C for 18–48 h. Following enrichment, subculture of Selenite F broth is made from the surface of the broth onto either MacConkey agar, Bismuth Sulfite agar, Deoxycholate Citrate agar, *Salmonella-Shigella* agar, Xylose-Lysine Deoxycholate agar or Hektoen Enteric agar, incubated at 37 °C for 24 h [45,46].

Some of the challenges in the application of culture-based approaches in isolating S. Typhi in endemic countries include the time taken to obtain culture results which takes at least 5–7 days, low sensitivity, lack of infrastructure and inadequate supply of trained manpower. In addition, another important limitation of culture method is the requirement of the use of antisera for confirmation of biochemical results indicating S. Typhi, which is resource-demanding for diagnostic laboratories in this setting.

### DNA Detection

Development of molecular tests for TF diagnosis requires genetic markers that are sensitive and specific for detection of bacterial DNA in blood of febrile patients [47]. Nucleic acid amplification tests, including conventional PCR, nested, multiplex and real-time PCR, have been developed for the detection of S. Typhi in blood [16,23,25,48,49,50,51]. Diagnostic markers which can detect pathogens at single-gene target resolution could lead to a simpler, cost-effective, and more functional DNA-based detection method since less primers are needed for target detection. Application of molecular techniques in clinical settings has technical limitations because of the few number of bacteria in blood, approximately 0.5 CFU/mL [16,51]. Molecular tests usually include primers targeting fliC-d in order to provide good specificity with little overlap with environmental isolates. Molecular detection of S. Typhi DNA in human blood has also been optimized through removal of background human DNA in order to improve the sensitivity of PCR for bacterial DNA and also reduce false positives. Enriching the PCR protocol to select for target bacterial DNA has been demonstrated to improve sensitivity by at least 1000-fold, compared to conventional methods of DNA preparation [52]. In addition, some researchers have further optimized molecular detection of S. Typhi DNA in human blood by inclusion of an incubation step. This technique described as blood culture-PCR, which includes a brief incubation step prior to PCR, has been developed in order to improve sensitivity and specificity of the assay. Blood samples of 5 mL were collected from controlled human infection models and incubated in ox-bile for 5 h, DNA extracted and amplified with primer targeted at S. Typhi fliC-d. This optimized molecular approach enabled the detection and confirmation of typhoid infection cases that would have been missed by blood culture [53]. Some of the limitations of molecular techniques include the identification of non-febrile patients with DNA in blood or bacteria shedding in stools leading to false positives. The main challenge with development of molecular assays is the applicability of these assays in resource-poor settings.

## 7. Serologic Testing

### 7.1. Widal Test

The Widal test was developed by Georges Ferdinand Widal in 1896 and helps to detect the presence of *Salmonella* antibodies in a patient’s serum. The Widal test measures agglutinating antibodies against the O and H antigens of S. Typhi in sera of people with suspected TF. Patients infected with *Salmonella* produce antibodies against the antigens of the organism. Antibodies in serum, produced in response to exposure to *Salmonella* antigens, will agglutinate bacterial suspension which carries homologous antigens. Antigens prepared from *Salmonella* are mixed with the patient’s serum to detect the presence of the antibodies. Agglutination reaction suggests a positive result, while absence suggests a negative result. In principle, to carry out the Widal test acute- and convalescent-phase serum samples should be collected approximately 10 days apart; a positive result is determined by a 4-fold increase in antibody titer [54]. However, antibody titers in infected patients often rise before the clinical onset of disease; this makes it challenging to demonstrate a 4-fold increase. In addition, in cases where patients supply paired sera, a decrease in titer is commonly observed when comparing the convalescent-phase serum to acute-phase serum. This might be due to the fact that most patients visit clinics during the convalescent phase, after initial pretreatments have failed. 

The Widal test is simple and inexpensive to perform, and widely used in developing countries, although it has limited diagnostic value. However, it suffers from a lack of standardization of reagents, poor specificity and inappropriate results interpretation [55]. Also, in areas of endemicity there is often a low background level of antibodies in the normal population, this makes it difficult to determine the appropriate cut-off point for a positive result due to differences between areas and between times [22]. Proper interpretation of Widal test results requires that each country determines the appropriate titer with which to diagnose typhoid.

### 7.2. Other Rapid Serologic Tests (Non-Widal)

Test characteristics of some commercially available RDTs are summarized in Table 1, but none of the RDTs have high sensitivity and/or specificity needed. Lack of specificity and sensitivity of Cromotest^®^—semiquantitative Slide agglutination, Tubex^®^, Cromotest^®^—single-tube Widal and Typhidot^®^ have been demonstrated elsewhere [21]. Other limitations of RDTs include limited application, difficulty in interpretation and affordability [56]. In the Philippines, the cost of a single test was as high as $51.68 for Tubex and $23.52 for Typhidot in some hospitals [29]. When compared with the Widal test which may not cost more than $1.5 per sample, these RDTs are not cost-effective and not affordable, especially in low-resource settings.

Tubex test, a colorimetric assay, might also pose difficulty in interpretation of hemolyzed samples. In addition, there might be false positives in patients infected with *S. enterica* serotype Enteritidis since Tubex detects immunoglobulin M directed towards S. Typhi O9 lipopolysaccharide antigen in sera. Other serological assays for TF that have been commercially developed include the Countercurrent Immuno electrophoresis test, which is based on the appearance of the precipitation band of antigen–antibody complexes that form on electrophoresis. The sensitivity is similar to that of the Widal test and the procedure may be quicker, but bands are often difficult to see and it is more expensive [57]. Additionally, several urine assays have been developed for TF testing although none has proved effective in detecting TF, despite the advantages of carrying out urine testing in resource-poor settings that are endemic for TF [58]. Generally, although RDTs have demonstrated some improvement over the Widal test, they still lack the required sensitivity, specificity, cost-effectiveness and consistency to allow their use as point-of-care diagnostics in endemic settings [59].

Available RDTs have variations in target antigens, for example, Typhidot detects specific IgM and IgG antibodies against the 50 kDa outer membrane protein (OMP). There is a modification of the Typhidot called Typhidot-M, detecting only IgM produced against the OMP, which is specific in acute infection. Additionally, because it detects only recent infections, it narrows the possibility of false-positive result due to previous infection. Multi-Test Dip-S-Ticks which detects five antibodies; the SD Bioline Typhoid rapid test, which uses an immunochromatographic method to detect IgG and IgM antibodies against an undefined *Salmonella* serovar typhi antigen; and a dipstick test named Enterocheck-WB that detects anti-LPS IgM antibodies [22,29,33]. SD Bioline, therefore, offers the advantages of detecting both chronic and acute infections, it is rapid (15–il min), and serum, plasma or even whole blood can be analyzed [59]. However, since detecting single antibody with single-antigen target have been demonstrated to increase specificity, multi-antibody approach may decrease specificity.

The abundance of IgG in the sera of people in highly endemic settings could limit the use of RDTs in these settings. For example, the use of Typhidot in highly endemic settings is limited by the abundance of IgG which can persist for more than 2 years [60], hence detection of specific IgG cannot differentiate between acute and convalescent cases. In order to mitigate the problem of sensitivity, an IgG depletion step was applied which led to the development of Typhidot-M which removed competitive binding and allowed accessibility of the antigen to the specific IgM [61].

### 7.3. Novel Biomarkers for Serodiagnosis

Serological markers currently available for typhoid diagnosis have a low degree of sensitivity and specificity. This necessitates the discovery of new targets that could be employed in serological analysis in the laboratory for accurate results. ELISAs have been developed and tested in detecting S. Typhi in endemic countries. A recent study in Nigeria tested IgA, IgM and IgG ELISAs using S. Typhi LPS and hemolysin E (HlyE) proteins on children with acute TF. The candidates for the ELISAs in the Nigerian setting was based on a proteome microarray data previously carried out by the same research group where they identified hemolysin E and LPS as putative biomarker targets. The receiver operator characteristic area under the curve (ROC-AUC) values suggested that LPS-specific IgA and IgA+M ELISA, in particular, was sensitive in diagnosing acute typhoid, and could discriminate well between typhoid and healthy, and other febrile bacteremias commonly encountered in Nigeria [62,63]. In another study in Bangladesh, 12 proteins were expressed and purified to design ELISAs and tested in a cohort of febrile patients. ELISAs were designed to detect IgM-specific antibodies to the 12 purified protein antigens and S. Typhi Vi polysaccharide antigen. Further analysis of ROC-AUC values revealed that the best three candidate antigens for serodiagnostics were encoded by STY4539 and STY1886 in combination with the Vi polysaccharide [64].

In a recent study, 4445 S. Typhi antigens were used to probe sera of individuals challenged with S. Typhi Quailes strains. Humoral immune responses were measured throughout duration of illness in the human challenge models. The study identified putative serodiagnostic biomarkers which include components of the bacterial cell surface (OmpA) and proteins targeted toward host cell attack (HlyE) and invasion (SipC) [65]. Other S. Typhi proteome array studies have indicated HlyE as a useful serodiagnostic marker based on IgA and IgG responses [62,65,66,67]. OmpA was also identified as a useful biomarker from proteomic screening [67] but is limited by being cross-reactive, expressed by other *Salmonella* and might not be a useful discriminator of S. Typhi infection in resource-poor settings where exposure to other *Salmonella* antigens might be frequent. S. Typhi proteome array screening has also identified two new putative biomarkers; *N*-acetylmuramoyl-l-alanine amidase (t2002, STY0927), which is involved in the catabolism of peptidoglycans and has previously been associated with invasion and intracellular survival of *Salmonella* Typhimurium and an uncharacterized hypothetical protein, t2295. Both putative protein targets gave IgM responses in controlled human infection models [64]. Inclusion of LPS into biomarker panels for S. Typhi screening seems to give better serodiagnostic value than panels without LPS as suggested in a study among pediatric children in Nigeria and in Nepal [62,65]. Antibody in Lymphocyte Supernatant assay (ALS) which has been used during vaccination and in other infections such as cholera [68], tuberculosis [69] and influenza [70] has also been applied to identify anti-S. Typhi antibodies in infected cohorts in Bangladesh [26]. More recently, ALS is being applied to decipher new serodiagnostic markers through immunoprofiling studies to identify immunodominant antigens that could be used in development of rapid serodiagnostics [67,71]. In all, recent S. Typhi proteome array studies suggest the need for inclusion of immunoglobulin targets specific to some of the putative proteins that have yielded better diagnostic value for evaluation in endemic settings. Some of the putative protein targets that should be considered for future diagnostic development for RDTs based on data from endemic countries are an IgA to S. Typhi LPS and IgG to HlyE for better diagnostic results in resource-limited settings [71]. These two targets have provided promising evidence of their usefulness as novel biomarkers for serodiagnosis in resource-poor settings endemic for S. Typhi infections.

## 8. Challenges in the Diagnosis of TF in Endemic Countries

TF is endemic in countries that are classified by the World Bank as Low-income or Low–middle-income countries (LMIC), or commonly referred to as developing countries. These countries are also plagued by other diseases such as malaria, tuberculosis and HIV, which compete for attention from the countries’ health sectors, in addition to several neglected tropical diseases. Poor laboratory infrastructure is a major impediment in TF-endemic countries. For example, in TF-endemic countries in Africa, most laboratories do not have the facilities and laboratory support to carry out blood culture, and the technical needs of bone marrow culture would make it very unlikely to be successfully performed in most of these settings. This implies that laboratories in resource-poor settings would usually rely on the poor diagnostic sensitivity of the Widal test for physicians to make clinical decisions on TF. In addition, there is no active surveillance system in place to get the actual estimates of disease burden, since most data available on TF are based on those that present to the hospital for treatment which would be a fraction of the actual number of cases, since pregnant women, neonates and those presenting to other departments in the hospital are not screened for TF. Under-estimation of the true disease burden has led to poor focus on TF, and reduced resource allocation in most affected countries [9]. Lack of rapid diagnostic tests that are affordable, very sensitive and specific has also hampered progress in TF diagnosis and treatment. Specimen collection remains an issue in TF diagnosis, as some of the laboratories do not have the manpower and infrastructure for timely collection and processing of blood samples in suspected patients. Over-diagnosis of TF in resource-limited settings owing to misuse of the Widal test has also resulted in the indiscriminate prescription of antibiotics. Furthermore, inappropriate antibiotic use due to their availability over the counter without isolation of the bacteria S. Typhi has contributed to the spread of Multidrug Resistant (MDR) S. Typhi. The emergence and spread of MDR *Salmonella* strains and decreasing efficacy of anti-typhoid drugs in endemic countries are huge sources of concern to public health stakeholders [72]. Chloramphenicol, Trimethoprim–sulphamethoxazole and ampicillin were the drugs of choice for many years for the treatment of typhoid fever, but are no longer in use in many countries because of the emergence of plasmid-mediated resistance about three decades ago [72,73]. To ensure better therapeutic results, fluoroquinolones, such as ciprofloxacin and ofloxacin, are still drugs of choice in endemic countries [57] instead of ceftriaxone due to their availability and affordability, even after the patent on these drugs expired in 2003 [73]. Unfortunately, strains of S. Typhi resistant to fluoroquinolones have emerged, causing increased treatment failure, disease severity, and even death [74,75]. Attempts to use vaccination in control of TF has also not been 100% effective. Vaccination targeting infants may not be sufficient to control the disease, since the epidemiology of TF suggests that a significant disease burden occurs in older populations beyond infancy and early childhood [76]. Three types of *Salmonella* vaccines are currently licensed; the oral live attenuated S. Typhi Ty21a vaccine, injectable Vi polysaccharide vaccine, and the recently approved Vi-tetanus toxoid (Vi-TT) conjugate vaccine [77,78]. However, these vaccines do not confer complete protection when administered, thus transmission and outbreaks are not fully preventable.

## 9. Conclusions

Without improved diagnostics, accurate estimates of the burden of typhoid, and planning by technical partners and governments concerned will remain challenging. There is a need for increased funding for laboratory infrastructure in developing countries especially in Sub-Saharan Africa where the true burden of typhoid is underappreciated, largely due to weak public health systems. While these efforts are being put in place, public health authorities in endemic countries should establish a threshold that would be used to determine positive Widal tests for proper interpretation of the most readily available, easy-to-perform and relatively noninvasive and affordable diagnostic, Widal in resource-poor settings. In addition, there should be a local surveillance data of enteric infections and other illnesses that cross-react with Salmonellae antigens in order to guide clinicians in interpretation of Widal test results. The approval by World Health Organization of the new Tybar-TCV^®^ vaccine suitable for children as young as 6 months, requiring a single dose with at least 55% protection, presents an opportunity for countries affected in Asia and Africa to incorporate TF vaccination into their routine childhood immunisation as an approach to reduce disease burden [79]. Lastly, there is an urgent need for rapid and improved diagnostics for accurate surveillance and monitoring impact of control efforts.

## Figures and Tables

**Table 1:** Summary of available Typhoid Fever Diagnostic Techniques.

Diagnostic Tests	Principle	Sensitivity	Specificity	References
Non-immunodiagnostic Methods				
Blood Culture	Based on the ability of viable cell to grow on culture medium	15.38–51.8%	100%	[18,19]
Stool Culture		<50%	93%	[20,21]
PCR (without enrichment in blood culture)	Relies on amplification of gene of interest	90–100%	100%	[22,23,24,25]
Immunodiagnostic Methods				
TPTest	Measures *S.* Typhi membrane preparation (MP)-specific IgA responses in peripheral blood mononuclear cell culture secretions	96.0%	96.6%	[19,26,27]
Tube Widal	Measures agglutinating antibodies against O and H antigens of *Salmonella* Typhi and *Salmonella* Paratyphi A; uses a tube or slide	65.38%	89.83%	[18,22]
Cromotest^®^ O: semiquantitative slide agglutination		95.2%	3.6%	[21]
Cromotest^®^ H: semiquantitative slide agglutination		80.3%	50.0	[21]
PanBio	ELISA detecting anti-LPS IgG and IgM	78%	80%	[28]
SD Bioline	ICT LFA cassette detecting IgG and IgM antibodies against an undefined *Salmonella* Typhi antigen	69%	79%	[29]
Mega *Salmonella*	ELISA detecting IgG and IgM antibodies against an undefined *Salmonella* Typhi antigen	91%	49%	[29]
LifeAssay Test-it	Detects IgM antibodies against *Salmonella typhi* LPS in an ICT LFA cassette	59%	98%	[30]
Typhidot	Measures IgM and IgG antibodies against a 50-kDa outer membrane protein of *Salmonella* Typhi in an immunodot test format	67–98%	58–100%	[29,31,32,33,34]
Typhidot M	Measures IgM antibodies, after removal of IgG antibodies, against a 50-kDa outer membrane protein of *Salmonella* Typhi in a dot blot format	47–98%	65–93%	[31,32,35]
TyphiRapid IgM and IgG IgM (Combo)	Measures IgM antibodies, after removal of IgG antibodies, against a 50-kDa outer membrane protein of *Salmonella* Typhi in an ICT LFA*a* cassette format	89–100%	85–89%	[36,37]
Tubex TF	Detects antibody against *Salmonella* Typhi LPS with an inhibition assay format and a visual result readout	56–100%	58–100%	[29,32,33,34,35,37,38]
Enterocheck-WB	Dipstick detecting anti-LPS IgM antibodies	89%	97%	[39]
Multi-Test-Dip-S-Ticks	Dipstick detecting anti-LPS IgG and IgM	89%	53%	[33]
PanBio	ELISA detecting anti-LPS IgG and IgM	78%	80%	[28]

## References

[1] Bitar R., Tarpley J. (1985). Intestinal perforation in typhoid fever: A historical and state-of-the-art review. Rev. Infect. Dis..

[2] Stevenson L.G. (1982). Exemplary disease: The typhoid pattern. J. Hist. Med. Allied Sci..

[3] Parry C.M., Hien T.T., Dougan G., White N.J., Farrar J.J. (2002). Typhoid fever. N. Engl. J. Med..

[4] Levine M.M., Black R.E., Lanata C. (1982). Precise estimation of the numbers of chronic carriers of Salmonella typhi in Santiago, Chile, an endemic area. J. Infect. Dis..

[5] Keddy K.H., Klugman K.P., Hansford C.F., Blondeau C., le Cam N.N.B. Persistence of Antibodies to the Salmonella Typhi Vi Capsular Polysaccharide Vaccine in South African School Children Ten Years after Immunization. PubMed-NCBI n.d. https://www.ncbi.nlm.nih.gov/pubmed/9987143/.

[6] Tauxe V., Tauxe M.D. (1991). Salmonella: A Postmodern Pathogen. J. Food Prot..

[7] Mogasale V., Maskery B., Ochiai R.L., Lee J.S., Mogasale V.V., Ramani E., Kim Y.E., Park J.K., Wierzba T.F. (2014). Burden of typhoid fever in low-income and middle-income countries: A systematic, literature-based update with risk-factor adjustment. Lancet Glob. Health.

[8] Mogasale V., Ramani E., Mogasale V.V., Park J. (2016). What proportion of Salmonella Typhi cases are detected by blood culture? A systematic literature review. Ann. Clin. Microbiol. Antimicrob..

[9] Obaro S.K., Iroh Tam P.-Y., Mintz E.D. (2017). The unrecognized burden of typhoid fever. Expert Rev. Vaccines.

[10] Crump J.A., Luby S.P., Mintz E.D. (2004). The global burden of typhoid fever. Bull World Health Organ..

[11] Kabwama S.N., Bulage L., Nsubuga F., Pande G., Oguttu D.W., Mafigiri R., Kihembo C., Kwesiga B., Masiira B., Okullo A.E. (2017). A large and persistent outbreak of typhoid fever caused by consuming contaminated water and street-vended beverages: Kampala, Uganda, January–June 2015. BMC Public Health.

[12] Egoz N., Shihab S., Leitner L., Lucian M. (1988). An outbreak of typhoid fever due to contamination of the municipal water supply in northern Israel. Isr. J. Med. Sci..

[13] Cutler D., Miller G. The Role of Public Health Improvements in Health Advances: The Twentieth-Century United States. PubMed-NCBI n.d. https://www.ncbi.nlm.nih.gov/pubmed/15782893/.

[14] Bennett S.D., Lowther S.A., Chingoli F., Chilima B., Kabuluzi S., Ayers T.L., Warne T.A., Mintz E. (2018). Assessment of water, sanitation and hygiene interventions in response to an outbreak of typhoid fever in Neno District, Malawi. PLoS ONE.

[15] Hornick R.B., Greisman S.E., Woodward T.E., DuPont H.L., Dawkins A.T., Snyder M.J. (1970). Typhoid fever: Pathogenesis and immunologic control. N. Engl. J. Med..

[16] Wain J., Diep T.S., Ho V.A., Walsh A.M., Nguyen T.T., Parry C.M., White N.J. (1998). Quantitation of bacteria in blood of typhoid fever patients and relationship between counts and clinical features, transmissibility, and antibiotic resistance. J. Clin. Microbiol..

[17] Juel H.B., Thomaides-Brears H.B., Darton T.C., Jones C., Jones E., Shrestha S., Sie R., Eustace A., Galal U., Kurupati P. (2017). Salmonella Typhi Bactericidal Antibodies Reduce Disease Severity but Do Not Protect against Typhoid Fever in a Controlled Human Infection Model. Front. Immunol..

[18] Maheshwari V., Kaore N.M., Ramnani V.K., Sarda S. (2016). A Comparative Evaluation of Different Diagnostic Modalities in the Diagnosis of Typhoid Fever Using a Composite Reference Standard: A Tertiary Hospital Based Study in Central India. J. Clin. Diagn. Res. JCDR.

[19] Islam K., Sayeed M.A., Hossen E., Khanam F., Charles R.C., Andrews J., Ryan E.T., Qadri F. (2016). Comparison of the Performance of the TPTest, Tubex, Typhidot and Widal Immunodiagnostic Assays and Blood Cultures in Detecting Patients with Typhoid Fever in Bangladesh, Including Using a Bayesian Latent Class Modeling Approach. PLoS Negl. Trop. Dis..

[20] Sattar A.A., Yusuf M.A., Islam M.B., Jahan W.A. (2014). Different Diagnostic Procedure of Typhoid Fever: A Review Update. J. Curr. Adv. Med. Res..

[21] Keddy K.H., Sooka A., Letsoalo M.E., Hoyland G., Chaignat C.L., Morrissey A.B., Crump J.A. Sensitivity and Specificity of Typhoid Fever Rapid Antibody Tests for Laboratory Diagnosis at Two Sub-Saharan African Sites. PubMed-NCBI n.d. https://www.ncbi.nlm.nih.gov/pubmed/21897484.

[22] Crump J.A., Sjölund-Karlsson M., Gordon M.A., Parry C.M. (2015). Epidemiology, Clinical Presentation, Laboratory Diagnosis, Antimicrobial Resistance, and Antimicrobial Management of Invasive Salmonella Infections. Clin. Microbiol. Rev..

[23] Ali A., Haque A., Haque A., Sarwar Y., Mohsin M., Bashir S., Tariq A. (2009). Multiplex PCR for differential diagnosis of emerging typhoidal pathogens directly from blood samples. Epidemiol. Infect..

[24] Chaudhry R., Chandel D.S., Verma N., Singh N., Singh P., Dey A.B. (2010). Rapid diagnosis of typhoid fever by an in-house flagellin PCR. J. Med. Microbiol..

[25] Song J.H., Cho H., Park M.Y., Na D.S., Moon H.B., Pai C.H. (1993). Detection of Salmonella typhi in the blood of patients with typhoid fever by polymerase chain reaction. J. Clin. Microbiol..

[26] Sheikh A., Bhuiyan M.S., Khanam F., Chowdhury F., Saha A., Ahmed D., Jamil K.M.A., LaRocque R.C., Harris J.B., Ahmad M.M. (2009). Salmonella enterica serovar Typhi-specific immunoglobulin A antibody responses in plasma and antibody in lymphocyte supernatant specimens in Bangladeshi patients with suspected typhoid fever. Clin. Vaccine Immunol. CVI.

[27] Khanam F., Sheikh A., Sayeed M.A., Bhuiyan M.S., Choudhury F.K., Salma U., Pervin S., Sultana T., Ahmed D., Goswami D. (2013). Evaluation of a Typhoid/Paratyphoid Diagnostic Assay (TPTest) Detecting Anti-Salmonella IgA in Secretions of Peripheral Blood Lymphocytes in Patients in Dhaka, Bangladesh. PLoS Negl. Trop. Dis..

[28] Gopalakrishnan V., Sekhar W.Y., Soo E.H., Vinsent R.A., Devi S. (2002). Typhoid fever in Kuala Lumpur and a comparative evaluation of two commercial diagnostic kits for the detection of antibodies to Salmonella typhi. Singapore Med. J..

[29] Kawano R.L., Leano S.A., Agdamag D.M.A. (2007). Comparison of serological test kits for diagnosis of typhoid fever in the Philippines. J. Clin. Microbiol..

[30] Pastoor R., Hatta M., Abdoel T.H., Smits H.L. (2008). Simple, rapid, and affordable point-of-care test for the serodiagnosis of typhoid fever. Diagn. Microbiol. Infect. Dis..

[31] Choo K.E., Davis T.M., Ismail A., Tuan Ibrahim T.A., Ghazali W.N. (1999). Rapid and reliable serological diagnosis of enteric fever: Comparative sensitivity and specificity of Typhidot and Typhidot-M tests in febrile Malaysian children. Acta Trop..

[32] Bhutta Z.A., Mansurali N. (1999). Rapid serologic diagnosis of pediatric typhoid fever in an endemic area: A prospective comparative evaluation of two dot-enzyme immunoassays and the Widal test. Am. J. Trop. Med. Hyg..

[33] Olsen S.J., Pruckler J., Bibb W., Nguyen T.M.T., Tran M.T., Nguyen T.M., Minh N.T., Sivapalasingam S., Gupta A., Phuong P.T. (2004). Evaluation of rapid diagnostic tests for typhoid fever. J. Clin. Microbiol..

[34] Naheed A., Ram P.K., Brooks W.A., Mintz E.D., Hossain M.A., Parsons M.M., Luby S.P., Breiman R.F. (2008). Clinical value of Tubex and Typhidot rapid diagnostic tests for typhoid fever in an urban community clinic in Bangladesh. Diagn. Microbiol. Infect. Dis..

[35] Dong B., Galindo C.M., Shin E., Acosta C.J., Page A.L., Wang M., Kim D., Ochiai R.L., Park J., Ali M. (2007). Optimizing typhoid fever case definitions by combining serological tests in a large population study in Hechi City, China. Epidemiol. Infect..

[36] Tarupiwa A., Tapera S., Mtapuri-Zinyowera S., Gumbo P., Ruhanya V., Gudza-Mugabe M., Majuru N.X., Chin’ombe N. (2015). Evaluation of TUBEX-TF and OnSite Typhoid IgG/IgM Combo rapid tests to detect *Salmonella enterica* serovar Typhi infection during a typhoid outbreak in Harare, Zimbabwe. BMC Res. Notes.

[37] Siba V., Horwood P.F., Vanuga K., Wapling J., Sehuko R., Siba P.M., Greenhill A.R. (2012). Evaluation of Serological Diagnostic Tests for Typhoid Fever in Papua New Guinea Using a Composite Reference Standard. Clin. Vaccine Immunol. CVI.

[38] House D., Wain J., Ho V.A., Diep T.S., Chinh N.T., Bay P.V., Vinh H., Duc M., Parry C.M., Dougan G. (2001). Serology of Typhoid Fever in an Area of Endemicity and Its Relevance to Diagnosis. J. Clin. Microbiol..

[39] Begum Z., Hossain M.A., Shamsuzzaman A.K.M., Ahsan M.M., Musa A.K.M., Mahmud M.C., Sumona A.A., Ahmed S., Jahan N.A., Khaleque M.A. (2009). Evaluation of Typhidot (IgM) for Early Diagnosis of Typhoid Fever. Bangladesh J. Med. Microbiol..

[40] Cheesbrough M. (2006). District Laboratory Practice in Tropical Countries.

[41] Cockerill F.R., Wilson J.W., Vetter E.A., Goodman K.M., Torgerson C.A., Harmsen W.S., Schleck C.D., Ilstrup D.M., Washington J., Wilson W.R. (2004). Optimal testing parameters for blood cultures. Clin. Infect. Dis. Off. Publ. Infect. Dis. Soc. Am..

[42] Wang S.K., Chu C.J., Sun P.S., Shan D.S., Kong F.L., Liu H.Y., Wu Q., Yang R.S., Yao Y.B. (2009). Study on blood cultures and bacteria counts in the blood of paratyphoid fever A patients. Eur. J. Clin. Microbiol. Infect. Dis. Off. Publ. Eur. Soc. Clin. Microbiol..

[43] Watson K.C. (1955). Isolation of Salmonella typhi from the Blood Stream. J. Lab. Clin. Med..

[44] Butler T., Bell W.R., Levin J., Linh N.N., Arnold K. (1978). Typhoid fever. Studies of blood coagulation, bacteremia, and endotoxemia. Arch. Intern. Med..

[45] World Health Organization (2003). The Diagnosis, Treatment and Prevention of Typhoid Fever.

[46] World Health Organization (2018). Bacterial Agents of Enteric Diseases of Public Health Concern 2018.

[47] Goay Y.X., Chin K.L., Tan C.L.L., Yeoh C.Y., Ja’afar J.N., Zaidah A.R., Chinni S.V., Phua K.K. (2016). Identification of Five Novel Salmonella Typhi-Specific Genes as Markers for Diagnosis of Typhoid Fever Using Single-Gene Target PCR Assays. BioMed Res. Int..

[48] Levy H., Diallo S., Tennant S.M., Livio S., Sow S.O., Tapia M., Fields P.I., Mikoleit M., Tamboura B., Kotloff K.L. (2008). PCR method to identify *Salmonella enterica* serovars Typhi, Paratyphi A, and Paratyphi B among Salmonella Isolates from the blood of patients with clinical enteric fever. J. Clin. Microbiol..

[49] Massi M.N., Shirakawa T., Gotoh A., Bishnu A., Hatta M., Kawabata M. (2003). Rapid diagnosis of typhoid fever by PCR assay using one pair of primers from flagellin gene of Salmonella typhi. J. Infect. Chemother. Off. J. Jpn. Soc. Chemother..

[50] Nga T.V.T., Karkey A., Dongol S., Thuy H.N., Dunstan S., Holt K., Campbell J.I., Chau T.T., Chau N.V.V., Arjyal A. (2010). The sensitivity of real-time PCR amplification targeting invasive Salmonella serovars in biological specimens. BMC Infect. Dis..

[51] Baker S., Favorov M., Dougan G. (2010). Searching for the elusive typhoid diagnostic. BMC Infect. Dis..

[52] Zhou L., Pollard A.J. (2012). A novel method of selective removal of human DNA improves PCR sensitivity for detection of Salmonella Typhi in blood samples. BMC Infect. Dis..

[53] Darton T.C., Zhou L., Blohmke C.J., Jones C., Waddington C.S., Baker S., Pollard A.J. (2017). Blood culture-PCR to optimise typhoid fever diagnosis after controlled human infection identifies frequent asymptomatic cases and evidence of primary bacteraemia. J. Infect..

[54] House D., Chinh N.T., Diep T.S., Parry C.M., Wain J., Dougan G., White N.J., Hien T.T., Farrar J.J. (2005). Use of paired serum samples for serodiagnosis of typhoid fever. J. Clin. Microbiol..

[55] Olopoenia L.A., King A.L. (2000). Widal agglutination test-100 years later: Still plagued by controversy. Postgrad Med. J..

[56] Kuijpers L.M.F., Chung P., Peeters M., Phoba M.-F., Kham C., Barbé B., Lunguya O., Jacobs J. (2018). Diagnostic accuracy of antigen-based immunochromatographic rapid diagnostic tests for the detection of Salmonella in blood culture broth. PLoS ONE.

[57] Wasfy M.O., Frenck R., Ismail T.F., Mansour H., Malone J.L., Mahoney F.J. Trends of Multiple-Drug Resistance among Salmonella Serotype Typhi Isolates During a 14-Year Period in Egypt. PubMed-NCBI n.d. https://www.ncbi.nlm.nih.gov/pubmed/12410488.

[58] Fadeel M.A., Crump J.A., Mahoney F.J., Nakhla I.A., Mansour A.M., Reyad B., El Melegi D., Sultan Y., Mintz E.D., Bibb W.F. (2004). Rapid diagnosis of typhoid fever by enzyme-linked immunosorbent assay detection of Salmonella serotype typhi antigens in urine. Am. J. Trop. Med. Hyg..

[59] Parry C.M., Wijedoru L., Arjyal A., Baker S. (2011). The utility of diagnostic tests for enteric fever in endemic locations. Expert Rev. Anti-Infect. Ther..

[60] Choo K.E., Davis T.M., Ismail A., Ong K.H. (1997). Longevity of antibody responses to a Salmonella typhi-specific outer membrane protein: Interpretation of a dot enzyme immunosorbent assay in an area of high typhoid fever endemicity. Am. J. Trop. Med. Hyg..

[61] Ismail A. (2000). New Advances in the Diagnosis of Typhoid and Detection of Typhoid Carriers. Malays J. Med. Sci..

[62] Huw Davies D., Jain A., Nakajima R., Liang L., Jasinskis A., Supnet M., Felgner P.L., Teng A., Pablo J., Molina D.M. (2016). Serodiagnosis of Acute Typhoid Fever in Nigerian Pediatric Cases by Detection of Serum IgA and IgG against Hemolysin E and Lipopolysaccharide. Am. J. Trop. Med. Hyg..

[63] Felgner J., Jain A., Nakajima R., Liang L., Jasinskas A., Gotuzzo E., Vinetz J.M., Miyajima F., Pirmohamed M., Hassan-Hanga F. (2017). Development of ELISAs for diagnosis of acute typhoid fever in Nigerian children. PLoS Negl. Trop. Dis..

[64] Tran Vu Thieu N., Trinh Van T., Tran Tuan A., Klemm E.J., Nguyen Ngoc Minh C., Voong Vinh P., Thanh D.P., Dan T.H.N., Duc T.P., Langat P. (2017). An evaluation of purified Salmonella Typhi protein antigens for the serological diagnosis of acute typhoid fever. J. Infect..

[65] Darton T.C., Baker S., Randall A., Dongol S., Karkey A., Voysey M., Carter M.J., Jones C., Trappl K., Pablo J. (2017). Identification of Novel Serodiagnostic Signatures of Typhoid Fever Using a Salmonella Proteome Array. Front. Microbiol..

[66] Liang L., Juarez S., Nga T.V.T., Dunstan S., Nakajima-Sasaki R., Davies D.H., McSorley S., Baker S., Felgner P.L. (2013). Immune profiling with a Salmonella Typhi antigen microarray identifies new diagnostic biomarkers of human typhoid. Sci. Rep..

[67] Charles R.C., Liang L., Khanam F., Sayeed M.A., Hung C., Leung D.T., Baker S., Ludwig A., Harris J.B., LaRocque R.C. (2014). Immunoproteomic analysis of antibody in lymphocyte supernatant in patients with typhoid fever in Bangladesh. Clin. Vaccine Immunol. CVI.

[68] Chang H.S., Sack D.A. (2001). Development of a novel in vitro assay (ALS assay) for evaluation of vaccine-induced antibody secretion from circulating mucosal lymphocytes. Clin. Diagn. Lab. Immunol..

[69] Raqib R., Rahman J., Kamaluddin A.K.M., Kamal S.M.M., Banu F.A., Ahmed S., Rahim Z., Bardhan P.K., Andersson J., Sack D.A. (2003). Rapid diagnosis of active tuberculosis by detecting antibodies from lymphocyte secretions. J. Infect. Dis..

[70] Halliley J.L., Kyu S., Kobie J.J., Walsh E.E., Falsey A.R., Randall T.D., Treanor J., Feng C., Sanz I., Lee F.E.H. (2010). Peak frequencies of circulating human influenza-specific antibody secreting cells correlate with serum antibody response after immunization. Vaccine.

[71] Darton T.C., Jones C., Dongol S., Voysey M., Blohmke C.J., Shrestha R., Karkey A., Shakya M., Arjyal A., Waddington C.S. (2017). Assessment and Translation of the Antibody-in-Lymphocyte Supernatant (ALS) Assay to Improve the Diagnosis of Enteric Fever in Two Controlled Human Infection Models and an Endemic Area of Nepal. Front. Microbiol..

[72] Rahman B.A., Wasfy M.O., Maksoud M.A., Hanna N., Dueger E., House B. (2014). Multi-drug resistance and reduced susceptibility to ciprofloxacin among Salmonella enterica serovar Typhi isolates from the Middle East and Central Asia. New Microbes New Infect..

[73] Butler T. (2011). Treatment of typhoid fever in the 21st century: Promises and shortcomings. Clin. Microbiol. Infect. Off. Publ. Eur. Soc. Clin. Microbiol. Infect. Dis..

[74] Antunes P., Mourão J., Campos J., Peixe L. (2016). Salmonellosis: The role of poultry meat. Clin. Microbiol. Infect. Off. Publ. Eur. Soc. Clin. Microbiol. Infect. Dis..

[75] Maurya P., Gulati A.K., Nath G. (2010). Status of Vi gene, its expression and Salmonella Pathogenicity Island (SPI-7) in Salmonella Typhi in India. Southeast Asian J. Trop. Med. Public Health.

[76] Atamanalp S.S., Aydinli B., Ozturk G., Oren D., Basoglu M., Yildirgan M.I. (2007). Typhoid intestinal perforations: Twenty-six year experience. World J. Surg..

[77] Galen J.E., Curtiss R. (2014). The delicate balance in genetically engineering live vaccines. Vaccine.

[78] Jin C., Gibani M.M., Moore M., Juel H.B., Jones E., Meiring J., Harris V., Gardner J., Nebykova A., Kerridge S.A. (2017). Efficacy and immunogenicity of a Vi-tetanus toxoid conjugate vaccine in the prevention of typhoid fever using a controlled human infection model of Salmonella Typhi: A randomised controlled, phase 2b trial. Lancet Lond. Engl..

[79] World Health Organization Typhoid Vaccine Prequalified 2018. http://www.who.int/medicines/news/2017/WHOprequalifies-breakthrough-typhoid-vaccine/en/.

